# Left ventricular postoperative false aneurysm following apical venting

**DOI:** 10.1186/1749-8090-1-41

**Published:** 2006-11-07

**Authors:** Federico Bizzarri, David Rose, Giacomo Frati, Luigi Muzzi

**Affiliations:** 1 Dipartimento Cuore e Grossi Vasi " Attilio Reale", Universita' degli Studi di Roma "La Sapienza", Unita' Operativa di Cardiochirurgia. Polo Pontino, Via F.Faggiana 34, Latina, Italy

## Abstract

We report a case of false aneurysm of the left ventricle occurring subsequently to the placing of a vent sump line through the apex during an aortic valve procedure; the diagnosis was made twelve months later during a routine echocardiographic examination. The lesion was successfully repaired. This case recommend the use of other routes of venting in order to reduce the incidence of such complications.

## Case report

**Figure 1:**
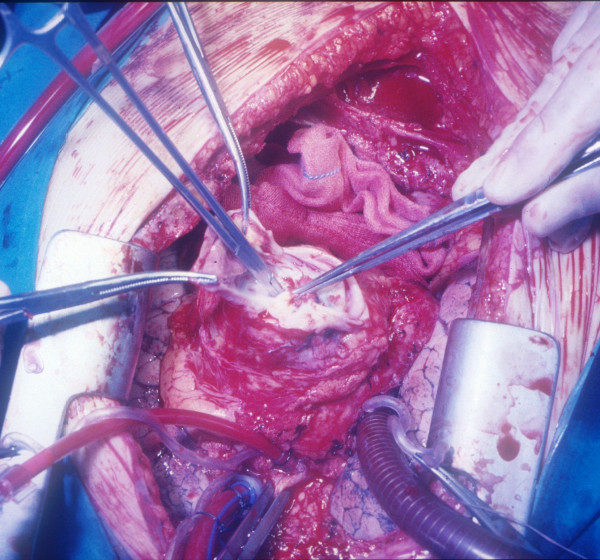
**The pseudoaneurysm mass as it appeared after isolation from epicardial adherences**. The pseudoaneurysm mass as it appeared after isolation from epicardial adherences (Figure 1). After the sac was opened (Figure 2), a leakage in the apical venting site was identified (Figure 3) and repaired with interrupted pledgeted polipropilene sutures (Figure 4).

**Figure 2:**
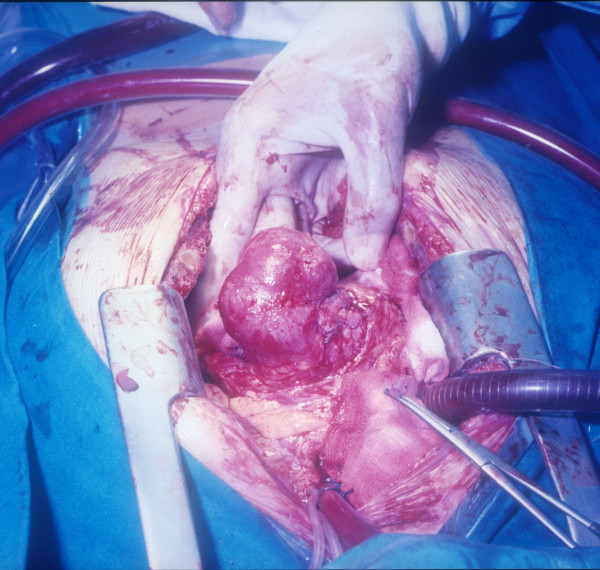
**The pseudoaneurysm mass after the sac was opened**. The pseudoaneurysm mass as it appeared after isolation from epicardial adherences (Figure 1). After the sac was opened (Figure 2), a leakage in the apical venting site was identified (Figure 3) and repaired with interrupted pledgeted polipropilene sutures (Figure 4).

**Figure 3:**
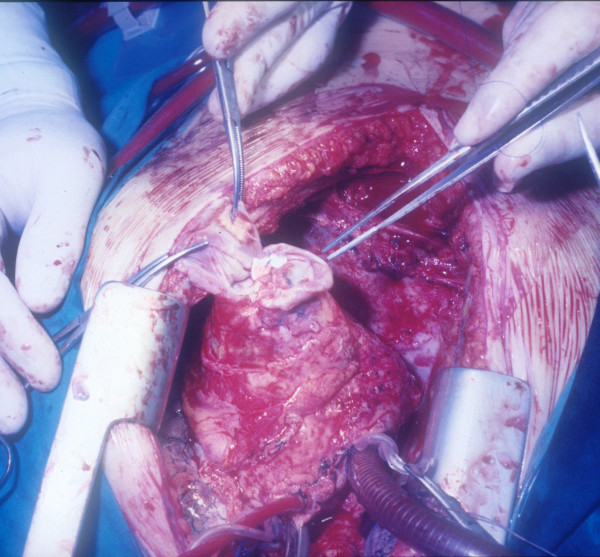
**A leakage in the apical venting site identified**. The pseudoaneurysm mass as it appeared after isolation from epicardial adherences (Figure 1). After the sac was opened (Figure 2), a leakage in the apical venting site was identified (Figure 3) and repaired with interrupted pledgeted polipropilene sutures (Figure 4).

**Figure 4:**
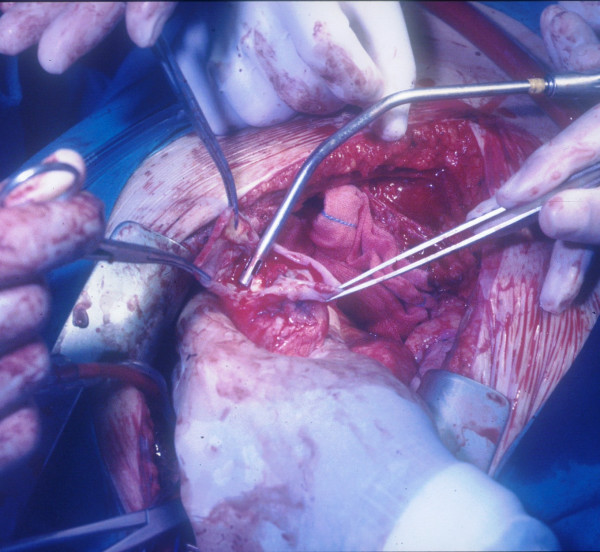
**The leakage repaired with interrupted pledgeted polipropilene sutures**. The pseudoaneurysm mass as it appeared after isolation from epicardial adherences (Figure 1). After the sac was opened (Figure 2), a leakage in the apical venting site was identified (Figure 3) and repaired with interrupted pledgeted polipropilene sutures (Figure 4).

Ventricular pseudoaneurysm is a complication of transmural myocardial infarction but may also follow blunt and penetrating chest trauma, inflammatory disease, infective endocarditis, tumours and cardiac surgery [1,2]. The pictures (Fig 1, 2, 3, 4) describe a case of false aneurysm of the left ventricle occurring as a consequence of placing a vent sump line through the apex during an aortic valve procedure. The procedure was accomplished with a mechanical prosthesis and the post operative course was uneventful. Twelve months later the patient underwent a routine echocardiographic evaluation; the four-chamber view revealed the presence of a cavity near the apex connecting to the left ventricle. The echocardiographic and radiologic examination indicated a pseudoaneurysm.

The lesion was successfully treated under cardiopulmonary by-pass and cardioplegic arrest. The mass was completely isolated, opened and identified as a false aneurysm. It appeared as a 5 cm × 3 cm red sac with free blood inside. The previous site of venting was identified and the cavity connected to the left ventricle (3 mm in diameter) closed with interrupted pledgeted polipropilene sutures. The patient was easily weaned off bypass and was discharged six days later.

Although effective, the apical venting can determine complications such as myocardial injury as well as serious difficulties in closing the vent site.

Because of the severity of the mentioned complication we recommend the use of different routes of left ventricular venting in order to prevent the risk of such complications.
